# Hapl-o-Mat: open-source software for HLA haplotype frequency estimation from ambiguous and heterogeneous data

**DOI:** 10.1186/s12859-017-1692-y

**Published:** 2017-05-30

**Authors:** Christian Schäfer, Alexander H. Schmidt, Jürgen Sauter

**Affiliations:** DKMS gemeinnützige GmbH, Kressbach 1, 72072 Tübingen, Germany

**Keywords:** HLA, Immunogenetics, Population genetics, Bioinformatics, Haplotype, Expectation-maximization algorithm, Open-source software

## Abstract

**Background:**

Knowledge of HLA haplotypes is helpful in many settings as disease association studies, population genetics, or hematopoietic stem cell transplantation. Regarding the recruitment of unrelated hematopoietic stem cell donors, HLA haplotype frequencies of specific populations are used to optimize both donor searches for individual patients and strategic donor registry planning. However, the estimation of haplotype frequencies from HLA genotyping data is challenged by the large amount of genotype data, the complex HLA nomenclature, and the heterogeneous and ambiguous nature of typing records.

**Results:**

To meet these challenges, we have developed the open-source software Hapl-o-Mat. It estimates haplotype frequencies from population data including an arbitrary number of loci using an expectation-maximization algorithm. Its key features are the processing of different HLA typing resolutions within a given population sample and the handling of ambiguities recorded via multiple allele codes or genotype list strings. Implemented in C++, Hapl-o-Mat facilitates efficient haplotype frequency estimation from large amounts of genotype data. We demonstrate its accuracy and performance on the basis of artificial and real genotype data.

**Conclusions:**

Hapl-o-Mat is a versatile and efficient software for HLA haplotype frequency estimation. Its capability of processing various forms of HLA genotype data allows for a straightforward haplotype frequency estimation from typing records usually found in stem cell donor registries.

**Electronic supplementary material:**

The online version of this article (doi:10.1186/s12859-017-1692-y) contains supplementary material, which is available to authorized users.

## Background

The use of current high-throughput genotyping technologies [1–4] provides information on alleles present at a locus of a diploid individual’s DNA, but not on the assignment of alleles along the same chromosome defining a haplotype. Knowledge of haplotypes of individuals from a population sample is important for inferring population evolutionary history [5]. Besides, haplotypes are examined in disease association studies to map patterns of genetic variation to diseases [6, 7]. In the context of unrelated hematopoietic stem cell transplantation (HSCT), population-specific human leukocyte antigen (HLA) haplotypes and their respective frequencies are of particular interest in strategic donor registry planning [8–11] and donor searches for individual patients using HLA matching algorithms [12–14].

Haplotypes can be inferred using genealogical information in families combined with targeted typing [15–17]. However, especially in large-scale studies this approach might not be feasible, as required information is not available or its provision is associated with additional costs. For instance, data as found in registries of unrelated potential HSCT donors generally lack information on family pedigrees. As an alternative, haplotype frequencies can be estimated from population-specific genotype data using a maximum likelihood estimation via an expectation-maximization (EM) algorithm [18–21].

Estimating HLA haplotype frequencies from potential HSCT donor registry typing records faces particular challenges. These challenges include large data sets, the complex HLA nomenclature [22], the heterogeneous nature of typing data in donor registries which originates from genotype data being recorded over extended periods of time using different strategies for applied typing resolution and typing profile [23], and genotyping ambiguities. Genotyping ambiguities result from typing techniques not being able to identify exactly two potentially different alleles at an individual’s specific HLA locus. Two types of genotyping ambiguities exist [24]: allelic and phase ambiguities. The former can occur when the nucleotide sequence is not completely examined, the latter when the chromosomal phase between polymorphisms cannot be established.

Typing results are recorded using designations assigned to HLA alleles by the WHO Nomenclature Committee for Factors of the HLA System [22]. These designations consist of up to four colon separated fields with digits which give information on the underlying nucleotide sequences. In HSCT, nucleotide sequences of exons encoding peptide and antigen binding domains are of particular importance [25]. HLA class I (class II) alleles with identical nucleotide sequences of exons 2 and 3 (exon 2 only) are summarized as G groups, whereas HLA class I (class II) alleles with identical amino acid sequences of exons 2 and 3 (exon 2 only) are summarized as P groups [22]. Alleles can also be summarized as g groups [26], which are defined analogous to P groups but include null alleles. The HLA nomenclature [22] provides HLA codes for P and G groups but not for g groups.

It has been shown that high-resolution (P group level) HLA matching is beneficial for transplantation outcome [27, 28]. The relevance of sequence differences outside the antigen-recognition domain (exons 2 and 3 for HLA class I, exon 2 for HLA class II) is still under debate [29]. A summary of typing resolutions and allele groups together with their definitions is shown in Table 1.

**Table 1:** Definitions of HLA typing resolutions and allele groups. For example, HLA alleles whose names share the same first two fields code for identical amino acid sequence. Hapl-o-Mat is able to translate between these typing resolutions and groups

Resolution/group	Definition
1 field	HLA alleles of identical allele group
2 fields	HLA alleles with identical amino acid sequences
3 fields^a^	HLA alleles with identical nucleotide sequences within the coding region
4 fields^a^	HLA alleles with identical nucleotide sequences within coding and non-coding (introns or 5' or 3' untranslated) regions
G	HLA class I (II) alleles with identical nucleotide sequences across exons 2 and 3 (2)
P	HLA class I (II) expressed alleles with identical amino acid sequences across exons 2 and 3 (2)
g	HLA class I (II) expressed and null alleles with identical amino acid sequences across exons 2 and 3 (2)

^a^Within the HLA nomenclature, 2 field designations comprise more field designations if the 2 field designation actually groups more than one allele, only. If the 2 field designation is already the full length designation, it is used as equivalent to 3 and 4 field designations in this paper

The National Marrow Donor Program (NMDP) has developed a broadly used system for reporting typing ambiguities by the introduction of HLA multiple allele codes known as NMDP codes [30]. If a typing yields an allelic ambiguity, all fields in the allele name except the first one are replaced by a letter code, currently comprising two to five letters, which encodes the possible alleles. Additionally, some NMDP codes represent alleles of different allele groups. However, since NMDP codes only consider information included in the first two fields, their use leads to a loss of information beyond the amino acid sequence. Furthermore, as NMDP codes do not include any phase information, phase ambiguities are transformed to and recorded as allelic ambiguities. This introduces new genotypes in addition to the original genotyping result [24]. An alternative to the NMDP code system are genotype list (GL) strings [24]. GL strings represent genotyping results including allelic and phase ambiguities without any coding-induced loss of information. P, G, and g groups are multiple allele codes as well. However, unlike GL strings and NMDP codes that impose no or virtually no restriction to members of a specific code, P, G, and g groups are only available as sets of alleles matching specific criteria (see Table 1).

Although several programs implement the EM algorithm for estimating HLA haplotype frequencies, none is able to entirely deal with the above mentioned challenges. One of the first freely available implementations of the EM algorithm was the software “Haplo” [31]. It handles incomplete typing data on some individuals and includes typing data from an individual’s relatives to complete or partially resolve the genotype. Additionally, it estimates errors on the derived haplotypes using a jackknife approach or the binomial standard error. The software “Arlequin” [32] supports different types of input and output data and includes several methods for population genetics data studies. It provides the standard EM algorithm and an extended version, the EM zipper algorithm, where haplotypes are reconstructed locus-wise. Furthermore, it supports the estimation of errors on derived haplotype frequencies using a bootstrap method. However, neither Haplo nor Arlequin are able to translate between different typing resolutions or to handle genotyping ambiguities. The software “Pypop” [33] includes several methods for performing population genetic analyses including the EM algorithm and focuses on analyses across many population data sets. With regard to the challenges found in potential HSCT donor registry typing records, Pypop checks HLA alleles in an input population for validity and can translate between typing resolutions of alleles but is currently restricted to a limited selection of possible translations. It is not capable of handling genotyping ambiguities. Besides various population genetics methods, the set of GENE[RATE] [34] programs includes a gene counting tool claimed to be equivalent to the EM algorithm to estimate haplotype frequencies. Optionally, the computation can include deviations from Hardy-Weinberg equilibrium (HWE) via an inbreeding coefficient. The tool is able to handle genotyping ambiguities. However, it does not support NMDP codes or GL strings but relies on its own syntax. Furthermore, translations of allelic to other typing resolutions are not supported. All GENE[RATE] tools are executed online via a web service, only.

To meet the challenges encountered in HLA haplotype frequency estimation from typical potential HSCT donor registry data, we developed the open-source software Hapl-o-Mat [35]. Hapl-o-Mat computes haplotype frequencies from population samples with arbitrary numbers of loci using an EM algorithm. Although it is not restricted to, it is specifically developed for HLA typing records (see Table 1). Thus, it has the functionality of translating between various typing resolutions of a given HLA gene. The result of an HLA gene typing in a given resolution can be expressed by its comprised alleles or by a G, P, or g group [22, 26] or it can be reduced to fewer fields in the allele name. Thus, typing records can be transformed to a uniform resolution rendering the typing resolution of input data for the EM algorithm homogeneous. The typing resolution is specified per locus by the user according to his needs. Furthermore, Hapl-o-Mat checks input data including HLA alleles for validity and processes genotyping ambiguities recorded as multiple allele codes (e.g. NMDP codes, G groups) or GL strings. Finally, its efficient implementation in C++ makes the estimation of haplotype frequencies from large data sets of up to millions of unphased genotypes feasible.

In the following, we review the EM algorithm and describe the implementation aspects Hapl-o-Mat uses to process genotype data and to estimate haplotype frequencies including translating between typing resolutions, resolving genotyping ambiguities, and initializing haplotype frequencies. We present Hapl-o-Mat validation results in terms of accurate haplotype frequency estimation using artificial data with known haplotype frequency distribution and comparisons with results provided by the software Arlequin [32]. Finally, we evaluate the computational performance of Hapl-o-Mat.

### Expectation-maximization algorithm

Haplotype frequencies can be estimated from population data using an EM algorithm. It computes the most probable set of haplotypes explaining the unphased genotype input data via a maximum likelihood estimation. Starting from arbitrary initial haplotype frequencies, it calculates genotype frequencies under the assumption of HWE (expectation step). After normalizing, these genotype frequencies are used to estimate haplotype frequencies (maximization step). Expectation and maximization steps are repeated until a stop criterion with predefined value is fulfilled.

The estimated likelihood is maximal within the precision of the stop criterion. However, the likelihood can reach multiple local maxima due to the non-linearity of the EM algorithm. The chance of arriving at a global maximum can be increased by running the EM algorithm several times with different initial haplotype frequencies.

## Implementation

**Fig. 1:**
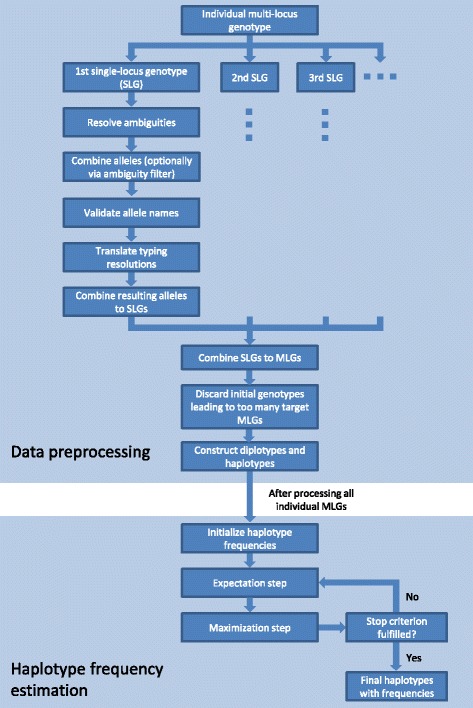
Workflow of Hapl-o-Mat. The main process is divided into data preprocessing and estimation of haplotype frequencies via the EM algorithm. The data preparation is illustrated for one individual MLG, which is split into several SLGs. After all individuals are processed, the estimation of haplotype frequencies starts. Expectation and maximization steps alternate until the stop criterion is fulfilled

The workflow of Hapl-o-Mat is divided into two major parts. First, Hapl-o-Mat preprocesses the input genotype data. This step includes resolving genotyping ambiguities and translating alleles to a uniform resolution per locus. Second, Hapl-o-Mat computes the most likely set of haplotypes including their frequencies via the EM algorithm. The workflow is illustrated in Fig. 1.

### Data preprocessing

Input data to Hapl-o-Mat is a population sample of genotype data. The data is read individual by individual and each multiple-locus genotype (MLG) is split into one genotype per locus (single-locus genotype (SLG)). The process of data preparation is exemplarily illustrated by two examples given in Additional file 1.

Hapl-o-Mat starts processing SLGs by resolving existing genotyping ambiguities. If the genotyping result was produced by Sanger sequencing-based typing, the number of resulting allele combinations can be reduced by applying an optional ambiguity filter. It only includes allele pairs that are possible but cannot be distinguished due to implicit ambiguities [36]. Otherwise, alleles are combined via a Cartesian product over both locus positions.

Next, alleles at the SLG are checked for validity. To this end, allele designations are compared to a list of all existing allele designations. This list is a copy of the allele designations database maintained by the WHO Nomenclature Committee for Factors of the HLA System [22] and is simply extracted by running a script before starting Hapl-o-Mat.

In order to deal with heterogeneous typing data, Hapl-o-Mat transforms SLGs to a uniform typing resolution. To this end, Hapl-o-Mat is capable of translating locus-wise between all typing resolutions and allele groups listed in Table 1. The translation process is explained in Additional file 2. If a translation yields several alleles per locus position, the alleles are combined via a Cartesian product over both locus positions.

Referring to the HLA nomenclature, a HLA typing with more fields contains more information on the underlying nucleotide sequence. However, translating typing results to a higher resolution is not associated with an information gain, since an expansion always includes all enclosed allele names equally weighted. On the other hand, translating to a lower resolution causes an information loss, due to the exclusion of fields from the allele designation.

After resolving genotyping ambiguities and translating to a uniform typing resolution, the resulting SLGs are combined to a set of MLGs using a Cartesian product. Thus, the original genotype from one individual can split into several genotypes of the envisaged target resolution. These are weighted by fractions summing up to one, as an individual actually only carries one genotype. If the initial genotype splits into a large amount of target genotypes, corresponding fractions can become small. As the effect of occasional low-weighted genotypes in haplotype frequency estimation is negligible [37, 38] and additional genotypes are computationally expensive in terms of speed and memory requirements, Hapl-o-Mat discards genotypes which split into more target resolution genotypes than a user-defined number from further analysis.

Finally, Hapl-o-Mat constructs diplotypes (pairs of haplotypes) and haplotypes from the resulting genotypes. These enter the second part of Hapl-o-Mat, the estimation of haplotype frequencies via the EM algorithm.

### Haplotype frequency estimation

Hapl-o-Mat computes the most likely set of haplotype frequencies accounting for the unphased input genotype data via an EM algorithm. It supports three different routines to initialize haplotype frequencies. First, frequencies are set to $1/{N}_{\mathrm{H}}$ with ${N}_H$ being the initial number of haplotypes. Second, frequencies are initialized according to numbers of occurrence of the respective haplotypes. Third, frequencies can be assigned randomly. The latter approach is implemented as adding a perturbation to frequencies initialized by the second method or as a completely random initialization. Random numbers are generated by a Mersenne Twister pseudorandom number generator [39].

After initialization, expectation and maximization steps are repeated until the maximal change in haplotype frequency between consecutive estimations is smaller than the stop criterion, a parameter specified by the user. After reaching the stop criterion, estimated haplotype frequencies smaller than a user-specified threshold are removed and, if specified by the user, the remaining haplotype frequencies are normalized. Eventually, inferred haplotypes with their respective frequencies are saved in an ASCII file format.

## Results and Discussion

We validated Hapl-o-Mat by checking its estimated haplotype frequencies for correctness. As translating between allele resolutions and resolving genotyping ambiguities are not supported by other software for haplotype frequency estimation, we followed two approaches. First, we validated Hapl-o-Mat against artificial HLA population data including different typing resolutions and genotyping ambiguities. For such artificial populations haplotype frequencies were known per construction. Taking the complete population data as an input sample, we used Hapl-o-Mat to resolve genotype data and to reproduce haplotype frequencies. Second, we compared results obtained from Hapl-o-Mat to results from the easy to use and well-established software Arlequin [32]. We used real samples of typing records from the DKMS donor center and artificial population data as input for both implementations. Furthermore, we evaluated the computational performance of Hapl-o-Mat in general and in comparison to Arlequin. The target resolution for all validation experiments are g groups unless noted otherwise.

For observables to compare haplotype frequencies and for the construction of artificial populations, see Methods in Additional file 3. All results are summarized in Table 2.

**Table 2:** Comparison of haplotype frequencies using distance$d$, maximal absolute difference between frequencies $\textcolor[rgb]{1,0,0}{\backslash}\textcolor[rgb]{1,0,0}{\mathrm{increment}}$, and first rank with a relative deviation larger than 0.05, $\rho$

Data	Remark	$d$	$\triangle$	$\rho$
First artificial population	Integer-valued genotype numbers	$1.3\times {10}^{-4}$	$9.04\times {10}^{-7}$	None
	Integer-valued genotype numbers and NMDP codes	$0.11\pm 0.02$	$\left(4\pm 1\right)\times {10}^{-3}$	$14\pm 6$
Second artificial population	Hapl-o-Mat – Population	$0.200$	$3.1\times {10}^{-3}$	$1$
	Arlequin – Population	$0.202$	$3.2\times {10}^{-3}$	$1$
	Arlequin – Hapl-o-Mat	$0.027$	$2.2\times {10}^{-4}$	$106$
Real genotype data	Hapl-o-Mat – Arlequin	$0.072\pm 0.002$	$\left(9\pm 2\right)\times {10}^{-4}$	$41\pm 23$

The observables were computed on basis of original and estimated haplotype frequencies. For the first artificial population, where we compared Hapl-o-Mat to population data, the column “Remark” indicates details of construction. For the other two genotype data sets, it indicates the sets of haplotype frequencies that are compared to each other, e.g. “Hapl-o-Mat – population” means haplotype frequencies obtained from Hapl-o-Mat were compared to original population haplotype frequencies

### First population model

The first artificial population was built by combinatorial construction of genotypes from all possible combinations of the $1,000$ most frequent German haplotypes with replacement, as explained in Additional file 3. The population was in almost perfect HWE as indicated by the effect size statistic ${W}_n=6.65\times {10}^{-8}$. To check translations between typing resolutions of Hapl-o-Mat, we replaced typing results with results in higher typing resolution including the original typing result, e.g. each occurrence of C*16:04 was randomly replaced by C*16:04:01, C*16:04:03, or C*16:04P or left unchanged as C*16:04. We used Hapl-o-Mat to translate the modified typing resolutions back to g groups and to estimate haplotype frequencies. The distance between estimated and original population haplotype frequencies was $d=1.3\times {10}^{-4}$, the maximal absolute difference was $\Delta =9.04\times {10}^{-7}$, and no relative deviation larger than 0.05 was found. These results indicated reproduction of the original population haplotype frequencies. Exact reproduction cannot not expected, as approximating genotype frequencies by integer numbers in the population data escapes floating point precision.

To validate the estimation of haplotype frequencies from genotype data including genotyping ambiguities, we introduced, in a second test, NMDP codes to the genotype population data. To this end, we randomly replaced 5% of typing results with NMDP codes. The codes were selected randomly except for the requirements to include the original typing and to have appeared in the original real population data. For example, all alleles typed as A*31:01 g were replaced with A*31:VSCB, which encodes A*31:01, A*31:41, and A*31:68 yielding two additional alleles (A*31:01 translates to A*31:01 g). Hapl-o-Mat with its ambiguity filter was used to resolve these ambiguities, translate the resulting alleles back to g groups, and compute haplotype frequencies. We repeated this procedure ten times to compute mean and standard deviation of observables.

Comparison between estimated and original population haplotype frequencies showed an average distance of $d=0.11\pm 0.02$, and an average maximal absolute difference of $\Delta =\left(4\pm 1\right)\times {10}^{-3}$. The average rank for the first haplotype with a relative deviation larger than 0.05 was $\rho =14\pm 6$. Compared to the first test, these larger values are explained by the occurrence of NMDP codes, which introduce additional alleles and thus mask real alleles. This obscures the identification of haplotypes by increasing the number of haplotypes not present in the original population set (“additional haplotypes”) and haplotypes only present in the original population set (“missing haplotypes”). The number of additional haplotypes is expected to be larger than the number of missing ones, since an NMDP code replaces only one allele but can yield several others when decoded. In the ten repetitions of the second test, on average $314\pm 98$ ($\left(25\pm 8\right)\%$) haplotypes were “additional” and $50\pm 18$ ($\left(4\pm 1\right)\%$) “missing”. These haplotypes made the major contribution to the difference between estimated and population haplotype frequencies. Excluding additional and missing haplotypes from computing the distance yielded $d=0.028\pm 0.007$.

**Fig. 2:**
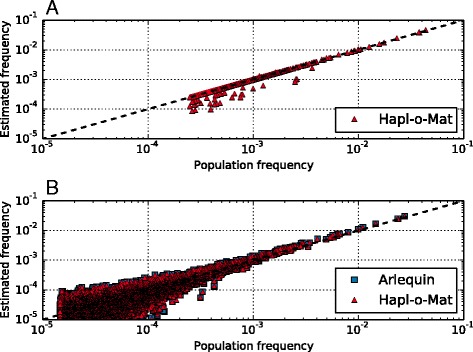
Haplotype frequencies from artificial population data. Plot **a** shows haplotype frequencies estimated via Hapl-o-Mat compared to original population frequencies from the first population model including genotyping ambiguities. Only one of ten runs is illustrated. Plot **b** shows a comparison between original population haplotype frequencies and frequencies estimated via Arlequin and Hapl-o-Mat on basis of the second population model. Due to the logarithmic scales, both plots neither show additional nor missing haplotypes

Original population and estimated frequencies are shown in Fig. 2a. As additional haplotypes have an original population frequency of ${h}_k=0$ and missing haplotypes have an estimated frequency of ${h}_k=0$, additional and missing haplotypes are not shown in Fig. 2a or in further log-log plots to come. Major deviations in haplotype frequencies were due to the occurrence of NMDP codes. If a haplotype included an allele which was masked by an NMDP code, its estimated frequency was reduced. If, on the other hand, a haplotype included additional alleles from an NMDP code, its estimated frequency increased. Only in few cases the frequency gain from additional alleles is transferred to haplotypes already present in the original population data. For this reason, almost no overestimation of haplotype frequencies (estimated frequency larger than original population frequency) occurs in Fig. 2a. However, the frequency loss from masked alleles belonging to haplotypes present in the original population data results in underestimation as found in Fig. 2a. Haplotypes which did not share alleles via NMDP codes only showed minor deviations between original population and estimated frequencies.

The fact that some estimated haplotype frequencies have a constant offset with regard to their original population frequency follows from sharing alleles found in the same NMDP code. The frequencies are reduced in proportion to the number of additional alleles emerging from the NMDP code. As a consequence, frequencies of haplotypes including alleles from the same NMDP code are reduced by the same factor.

### Second population model

The second population was built by constructing genotypes from randomly combining two haplotypes according to their frequency distribution as explained in Additional file 3. The effect size statistic averaged over all loci for this population was ${W}_n=3.0\times {10}^{-3}$ indicating no significant devation from HWE. We computed haplotype frequencies from these population data using Arlequin and Hapl-o-Mat. The estimated and original population haplotype frequencies are shown in Fig. 2b. The corresponding observables are given in Table 2. Both implementations performed equally well demonstrating the correct implementation of Hapl-o-Mat. However, in contrast to the first population model, deviations between estimated and original population frequencies were much larger both for Arlequin and Hapl-o-Mat. This resulted from applying the EM algorithm to data with a large amount of genotype diversity. As the data consisted of only $N=50,000$ individuals but included $41,489$ different genotypes, the EM algorithm was not able to exactly reproduce the original population haplotype frequency distribution. For this reason Arlequin and Hapl-o-Mat, both based on the EM algorithm, showed similar deviations between estimated and original population frequencies as observed in Fig. 2b.

#### Real data samples

Finally, we estimated haplotype frequencies from real population data. Ten samples of $N=50,000$ individuals were drawn from $N=1,825,721$ individuals of self-assessed German origin registered with DKMS donor center and typed for HLA-A, -B, -C, -DRB1, -DQB1, and -DPB1. We only included typing results translating unambiguously to 2-field resolution in order to be able to include Arlequin into analysis. By averaging over ten samples, we give mean and standard deviation of each observable. The effect size statistic averaged over all loci and samples was ${W}_n=\left(2.1\pm 0.4\right)\times {10}^{-3}$ indicating no significant deviation from HWE.

**Fig. 3:**
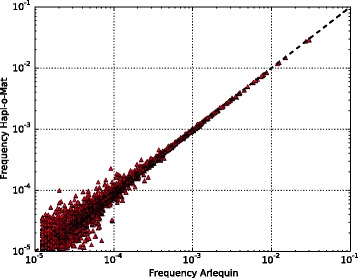
Comparison of haplotype frequencies estimated via Arlequin and Hapl-o-Mat from one sample of real population data. Due to the logarithmic scales, the plot neither shows additional nor missing haplotypes

Comparing resulting haplotype frequencies between Arlequin and Hapl-o-Mat, the distance was ${d}_{\mathrm{Arlequin}}^{\mathrm{Haplomat}}=0.072\pm 0.002$, the maximal absolute difference between frequencies was ${\Delta}_{\mathrm{Arlequin}}^{\mathrm{Haplomat}}=\left(9\pm 2\right)\times {10}^{-4}$, and the first rank with a relative deviation larger than 0.05 was ${\rho}_{\mathrm{Arlequin}}^{\mathrm{Haplomat}}=41\pm 23$. These values were of similar magnitude as results from comparing Arlequin to Hapl-o-Mat on basis of the second artificial population model, see Table 2, indicating a correct implementation of Hapl-o-Mat. The similarity of estimated haplotype frequencies is depicted in Fig. 3.

### Computational performance

We evaluated Hapl-o-Mat in terms of computational performance by measuring its runtime for different amounts of input data and different target resolutions. All computations were performed using a computer running Ubuntu Linux 14.04.5 with 768 GB RAM (although this was never exhausted), and 32 Intel® Xeon® CPU E5-2630 v3 cores at 2.40GHz. However, Hapl-o-Mat does not make use of parallelism, hence all runtime are in reference to a single core.

The runtime for estimating haplotype frequencies by Hapl-o-Mat from N=1,825,721 individuals with self-assessed German origin was $t\approx 11.4$h with g groups as target resolution.

**Fig. 4:**
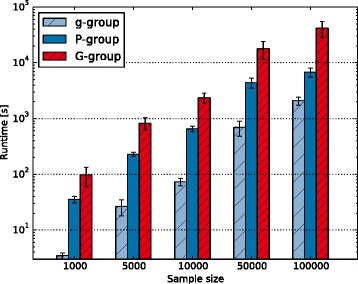
Average runtimes with standard deviation of Hapl-o-Mat for different sample sizes and different target allele groups including g, P, and G groups

We further drew random subsamples of sizes $N=1,000$, $N=5,000$, $N=10,000$, $N=50,000$, and $N=100,000$individuals. For more information on the composition of these data please refer to Additional file 3. The sampling process was repeated ten times per sample size and target resolution to compute average times for running Hapl-o-Mat. The target resolution was varied between g, P, and G groups. Hapl-o-Mat was run with activated normalization, without ambiguity filter, and starting from perturbed initial haplotype frequencies. The runtimes are illustrated in Fig. 4.

In order to compare the performance between Arlequin and Hapl-o-Mat, we repeated the haplotype frequency estimation from real population data. We varied the sample size between $N=5,000$, $N=20,000$, and $N=50,000$ and similarly included only samples with unambiguous 2-field translation. Averaging both implementations over ten runs on the same machine yielded runtimes as given in Table 3. Especially in the case of large sample sizes, Hapl-o-Mat was considerably faster demonstrating its efficient implementation.

**Table 3:** Average runtimes of Arlequin and Hapl-o-Mat for estimation of haplotype frequencies from real population data

Sample size	Runtime Arlequin [s]	Runtime Hapl-o-Mat [s]	Ratio
5000	$242\pm 4$	$10\pm 2$	$24\pm 5$
20000	$6617\pm 971$	$39\pm 4$	$170\pm 30$
50000	$40539\pm 2,383$	$83\pm 10$	$488\pm 65$

**Fig. 5:**
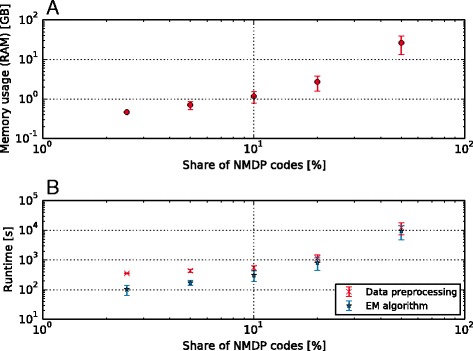
Performance of Hapl-o-Mat with regard to varying share of typing records containing NMDP codes. Plot **a** shows average memory usage with standard deviations and Plot **b** average runtimes with standard deviations for both; data preprocessing and haplotype frequency estimation

We also evaluated Hapl-o-Mat’s abilities to cope with the heterogeneous and ambiguous nature of typing records. We recorded runtime and memory usage on the machine described above as we varied the share of NMDP codes we introduced in the genotype population data for the first population model in the same manner as described above for a varying fraction of masked alleles from 2.5% to 50%. Hapl-o-Mat with its ambiguity filter was used to resolve these ambiguities, translate the resulting alleles back to g groups, and compute haplotype frequencies. We repeated this procedure ten times to compute mean and standard deviation of memory usages and runtimes. The results are visualized in Fig. 5.

## Conclusions

We have presented Hapl-o-Mat, an open-source software for HLA haplotype frequency estimation. It is the first publically available software that meets the challenges encountered in hematopoietic stem cell donor registry data. It supports translations between typing resolutions, is capable of resolving genotyping ambiguities, and handles large-scale HLA genotype data, due to its efficient implementation in C++. Its conjunction of data preprocessing and EM algorithm in one software offers a straightforward way of haplotype frequency estimation from HLA population data.

## Additional files

Additional file 1: Examples for Data Preprocessing. (PDF 468 kb)

Additional file 2: Translation between Typing Resolutions. (PDF 303 kb)

Additional file 3: Methods. (PDF 609 kb)

## Abbreviations

EM: expectation-maximization
GL: genotype list
HF: haplotype frequency
HLA: human leukocyte antigen
HSCT: hematopoietic stem cell transplantation
HWE: Hardy-Weinberg equilibrium
MLG: multiple-locus genotype
NMDP: National marrow donor program
SLG: single-locus genotype

## References

[1] Bentley G, Higuchi R, Hoglund B, Goodridge D, Sayer D, Trachtenberg EA, Erlich HA (2009). High-resolution, high-throughput HLA genotyping by next-generation sequencing. Tissue Antigens.

[2] Lind C, Ferriola D, Mackiewicz K, Heron S, Rogers M, Slavich L, Walker R, Hsiao T, McLaughlin L, D'Arcy M (2010). Next-generation sequencing: the solution for high-resolution, unambiguous human leukocyte antigen typing. Hum Immunol.

[3] Lange V, Böhme I, Hofmann J, Lang K, Sauter J, Schöne B, Paul P, Albrecht V, Andreas JM, Baier DM (2014). Cost-efficient high-throughput HLA typing by MiSeq amplicon sequencing. BMC Genomics.

[4] Schofl G, Lang K, Quenzel P, Bohme I, Sauter J, Hofmann JA, Pingel J, Schmidt AH, Lange V (2017). 2.7 million samples genotyped for HLA by next generation sequencing: lessons learned. BMC Genomics.

[5] Harding RM, Fullerton SM, Griffiths RC, Bond J, Cox MJ, Schneider JA, Moulin DS, Clegg JB (1997). Archaic African and Asian lineages in the genetic ancestry of modern humans. Am J Hum Genet.

[6] Risch N, Merikangas K (1996). The future of genetic studies of complex human diseases. Science.

[7] Crawford DC, Nickerson DA (2005). Definition and clinical importance of haplotypes. Annu Rev Med.

[8] Beatty PG, Dahlberg S, Mickelson EM, Nisperos B, Opelz G, Martin PJ, Hansen JA (1988). Probability of finding HLA-matched unrelated marrow donors. Transplantation.

[9] Hurley CK, Fernandez Vina M, Setterholm M (2003). Maximizing optimal hematopoietic stem cell donor selection from registries of unrelated adult volunteers. Tissue Antigens.

[10] Schmidt AH, Solloch UV, Baier D, Stahr A, Wassmuth R, Ehninger G, Rutt C (2010). Regional differences in HLA antigen and haplotype frequency distributions in Germany and their relevance to the optimization of hematopoietic stem cell donor recruitment. Tissue Antigens.

[11] Schmidt AH, Sauter J, Pingel J, Ehninger G (2014). Toward an optimal global stem cell donor recruitment strategy. PLoS ONE.

[12] Eberhard HP, Feldmann U, Bochtler W, Baier D, Rutt C, Schmidt AH, Muller CR (2010). Estimating unbiased haplotype frequencies from stem cell donor samples typed at heterogeneous resolutions: a practical study based on over 1 million German donors. Tissue Antigens.

[13] Steiner D (2012). Computer algorithms in the search for unrelated stem cell donors. Bone Marrow Res.

[14] Bochtler W, Gragert L, Patel ZI, Robinson J, Steiner D, Hofmann JA, Pingel J, Baouz A, Melis A, Schneider J (2016). A comparative reference study for the validation of HLA-matching algorithms in the search for allogeneic hematopoietic stem cell donors and cord blood units. HLA.

[15] Perlin MW, Burks MB, Hoop RC, Hoffman EP (1994). Toward fully automated genotyping: allele assignment, pedigree construction, phase determination, and recombination detection in Duchenne muscular dystrophy. Am J Hum Genet.

[16] Becker T, Knapp M (2002). Efficiency of haplotype frequency estimation when nuclear family information is included. Hum Hered.

[17] Ikeda N, Kojima H, Nishikawa M, Hayashi K, Futagami T, Tsujino T, Kusunoki Y, Fujii N, Suegami S, Miyazaki Y (2015). Determination of HLA-A, -C, -B, -DRB1 allele and haplotype frequency in Japanese population based on family study. Tissue Antigens.

[18] Dempster AP, Laird NM, Rubin DB (1977). Maximum Likelihood from Incomplete Data via the EM Algorithm. J R Stat Soc Ser B (Methodological).

[19] Excoffier L, Slatkin M (1995). Maximum-likelihood estimation of molecular haplotype frequencies in a diploid population. Mol Biol Evol.

[20] Long JC, Williams RC, Urbanek M (1995). An E-M algorithm and testing strategy for multiple-locus haplotypes. Am J Hum Genet.

[21] Polańska J (2003). The EM algorithm and its implementation for the estimation of frequencies of SNP-haplotypes. Int J Appl Marth Comp Sci.

[22] Marsh SGE, Albert ED, Bodmer WF, Bontrop RE, Dupont B, Erlich HA, Fernandez-Vina M, Geraghty DE, Holdsworth R, Hurley CK (2010). Nomenclature for factors of the HLA system, 2010. Tissue Antigens.

[23] Sauter J, Solloch UV, Giani AS, Hofmann JA, Schmidt AH (2016). Simulation shows that HLA-matched stem cell donors can remain unidentified in donor searches. Sci Rep.

[24] Milius RP, Mack SJ, Hollenbach JA, Pollack J, Heuer ML, Gragert L, Spellman S, Guethlein LA, Trachtenberg EA, Cooley S (2013). Genotype List String: a grammar for describing HLA and KIR genotyping results in a text string. Tissue Antigens.

[25] Copelan EA (2006). Hematopoietic stem-cell transplantation. N Engl J Med.

[26] Schmidt AH, Baier D, Solloch UV, Stahr A, Cereb N, Wassmuth R, Ehninger G, Rutt C (2009). Estimation of high-resolution HLA-A, -B, -C, -DRB1 allele and haplotype frequencies based on 8862 German stem cell donors and implications for strategic donor registry planning. Hum Immunol.

[27] Lee SJ, Klein J, Haagenson M, Baxter-Lowe LA, Confer DL, Eapen M, Fernandez-Vina M, Flomenberg N, Horowitz M, Hurley CK (2007). High-resolution donor-recipient HLA matching contributes to the success of unrelated donor marrow transplantation. Blood.

[28] Eapen M, Klein JP, Ruggeri A, Spellman S, Lee SJ, Anasetti C, Arcese W, Barker JN, Baxter-Lowe LA, Brown M (2014). Impact of allele-level HLA matching on outcomes after myeloablative single unit umbilical cord blood transplantation for hematologic malignancy. Blood.

[29] Hou L, Vierra-Green C, Lazaro A, Brady C, Haagenson M, Spellman S, Hurley CK (2017). Limited HLA sequence variation outside of antigen recognition domain exons of 360 10 of 10 matched unrelated hematopoietic stem cell transplant donor-recipient pairs. Hla.

[30] Allele Code Lists [https://bioinformatics.bethematchclinical.org/HLA-Resources/Allele-Codes/Allele-Code-Lists/]. Accessed 25 May 2017.

[31] Hawley ME, Kidd KK (1995). HAPLO: a program using the EM algorithm to estimate the frequencies of multi-site haplotypes. J Hered.

[32] Excoffier L, Lischer HE (2010). Arlequin suite ver 3.5: a new series of programs to perform population genetics analyses under Linux and Windows. Mol Ecol Resour.

[33] Lancaster AK, Single RM, Solberg OD, Nelson MP, Thomson G (2007). PyPop update--a software pipeline for large-scale multilocus population genomics. Tissue Antigens.

[34] Nunes JM, Buhler S, Roessli D, Sanchez-Mazas A, collaboration HL-n (2014). The HLA-net GENE[RATE] pipeline for effective HLA data analysis and its application to 145 population samples from Europe and neighbouring areas. Tissue Antigens.

[35] Hapl-o-Mat: A software for haplotype inference [https://github.com/DKMS/Hapl-o-Mat]. Accessed 25 May 2017.

[36] Robinson J, Halliwell JA, Hayhurst JD, Flicek P, Parham P, Marsh SG (2015). The IPD and IMGT/HLA database: allele variant databases. Nucleic Acids Res.

[37] Gragert L, Madbouly A, Freeman J, Maiers M (2013). Six-locus high resolution HLA haplotype frequencies derived from mixed-resolution DNA typing for the entire US donor registry. Hum Immunol.

[38] Pingel J, Solloch UV, Hofmann JA, Lange V, Ehninger G, Schmidt AH (2013). High-resolution HLA haplotype frequencies of stem cell donors in Germany with foreign parentage: how can they be used to improve unrelated donor searches?. Hum Immunol.

[39] Matsumoto M, Nishimura T (1998). Mersenne twister: a 623-dimensionally equidistributed uniform pseudo-random number generator. ACM Trans Model Comput Simul.

