# Supplementary material for: Hapl-o-Mat: open-source software for HLA haplotype frequency estimation from ambiguous and heterogeneous data
Source: BMC Bioinformatics. 2017 May 30;18:284. doi: 10.1186/s12859-017-1692-y (PMC5450239; doi:10.1186/s12859-017-1692-y)
Supplement: Supplementary file 1 — Examples for Data Preprocessing. (PDF 468 kb) [file 12859_2017_1692_MOESM1_ESM.pdf]

Additional File 1 – Examples for Data Preprocessing

We give two examples on the data preprocessing of Hapl-o-Mat. Data preprocessing starts with splitting the initial genotype of one individual into SLGs and these into alleles and NMDP codes. At this level, every HLA assignment or NMDP code has a frequency of $f = 1$, as indicated by the light blue boxes in the examples. Next, existing NMDP codes are resolved to their enclosed alleles. After this step, all alleles are validated against a list of the allele name database maintained by the WHO Nomenclature Committee for Factors of the HLA System. If the allele is not found in this list, Hapl-o-Mat discards the full genotype. Else, alleles are translated to a resolution corresponding to a full-length designation and then to the target resolution. The translation process is explained in Additional File 2. The resulting alleles are combined to SLGs via a Cartesian product over both locus fields. In turn, the resulting SLGSs are combined to a final set of MLGs in target resolution. In the end, the sum of resulting genotype weights is $\sum_i f_i = 1$.

In the first example, we process the genotype

A*01:01:01+A*01:67^B*27:AB+B*13:02:04

and in the second example the genotype

A*24:BMD+A*33:01:01^DQB1*06:FTZK+DQB1*06:HTC.

In both cases, we translate the typing resolution at locus HLA-A to G groups and at locus HLA-B to g groups. The underlying version of the IMGT/HLA database is 3.23 in all examples.

Example One

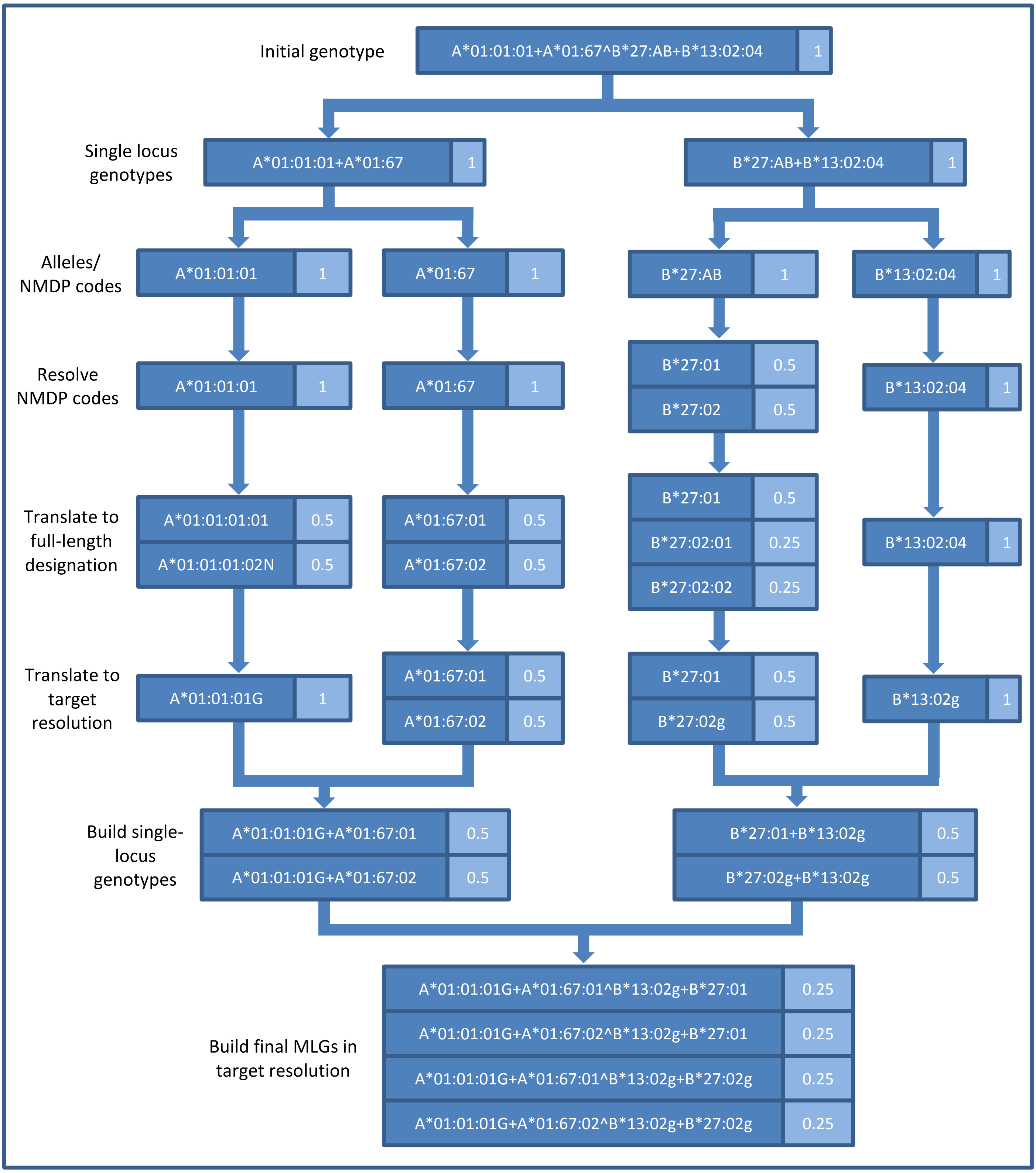

Initial genotype
A*01:01:01+A*01:67^B*27:AB+B*13:02:04
1
Single locus genotypes
A*01:01:01+A*01:67
1
B*27:AB+B*13:02:04
1
Alleles/ NMDP codes
A*01:01:01
1
A*01:67
1
B*27:AB
1
B*13:02:04
1
Resolve NMDP codes
A*01:01:01
1
A*01:67
1
B*27:01
0.5
B*27:02
0.5
B*13:02:04
1
Translate to full-length designation
A*01:01:01:01
0.5
A*01:01:01:02N
0.5
A*01:67:01
0.5
A*01:67:02
0.5
B*27:01
0.5
B*27:02:01
0.25
B*27:02:02
0.25
B*13:02:04
1
Translate to target resolution
A*01:01:01G
1
A*01:67:01
0.5
A*01:67:02
0.5
B*27:01
0.5
B*27:02g
0.5
B*13:02g
1
Build single-locus genotypes
A*01:01:01G+A*01:67:01
0.5
A*01:01:01G+A*01:67:02
0.5
B*27:01+B*13:02g
0.5
B*27:02g+B*13:02g
0.5
Build final MLGs in target resolution
A*01:01:01G+A*01:67:01^B*13:02g+B*27:01
0.25
A*01:01:01G+A*01:67:02^B*13:02g+B*27:01
0.25
A*01:01:01G+A*01:67:01^B*13:02g+B*27:02g
0.25
A*01:01:01G+A*01:67:02^B*13:02g+B*27:02g
0.25

Example Two

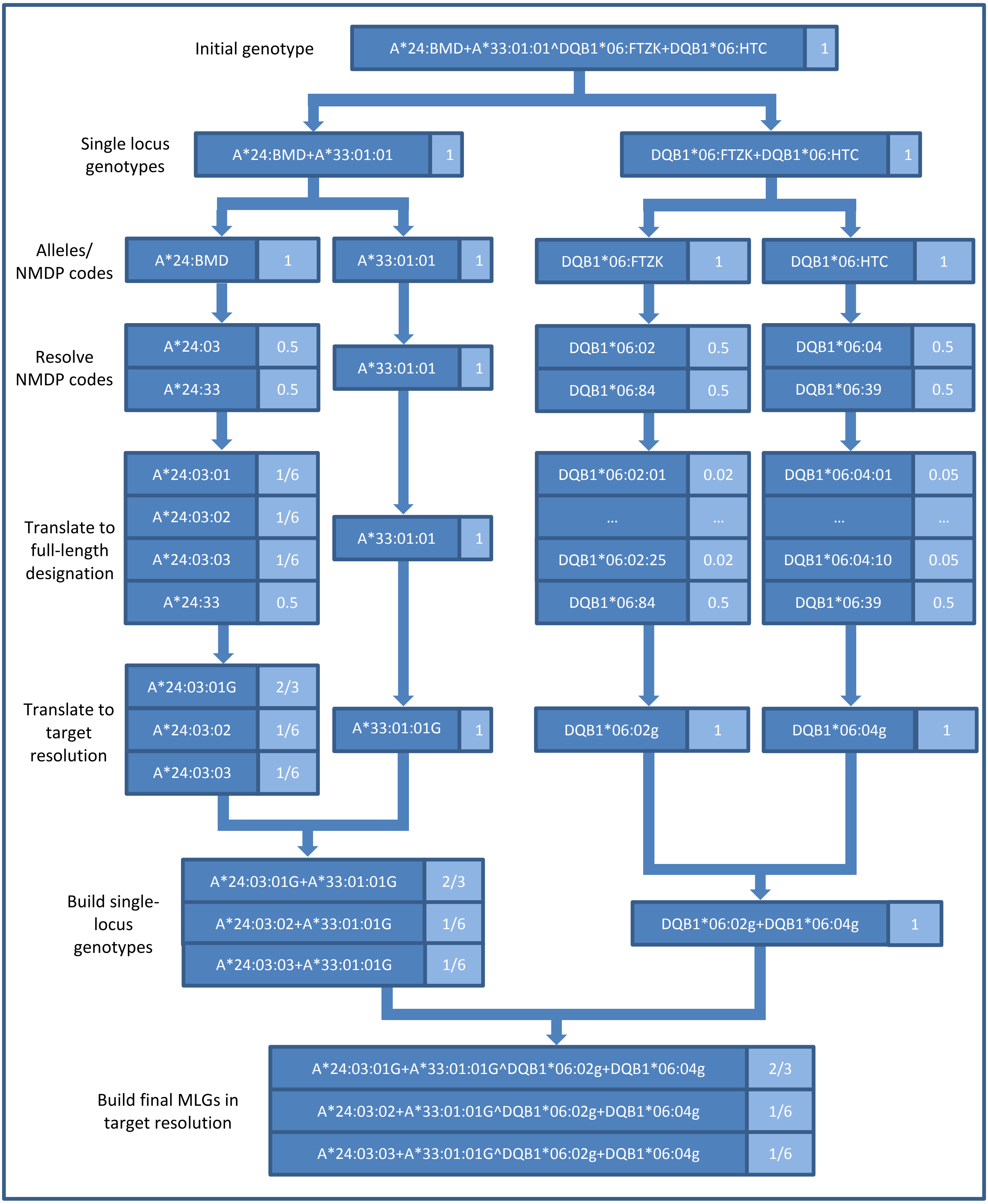

Initial genotype
A*24:BMD+A*33:01:01^DQB1*06:FTZK+DQB1*06:HTC
1
Single locus genotypes
A*24:BMD+A*33:01:01
1
DQB1*06:FTZK+DQB1*06:HTC
1
Alleles/ NMDP codes
A*24:BMD
1
A*33:01:01
1
DQB1*06:FTZK
1
DQB1*06:HTC
1
Resolve NMDP codes
A*24:03
0.5
A*24:33
0.5
A*33:01:01
1
DQB1*06:02
0.5
DQB1*06:84
0.5
DQB1*06:04
0.5
DQB1*06:39
0.5
Translate to full-length designation
A*24:03:01
1/6
A*24:03:02
1/6
A*24:03:03
1/6
A*24:33
0.5
A*33:01:01
1
DQB1*06:02:01
0.02
...
...
DQB1*06:02:25
0.02
DQB1*06:84
0.5
DQB1*06:04:01
0.05
...
...
DQB1*06:04:10
0.05
DQB1*06:39
0.5
Translate to target resolution
A*24:03:01G
2/3
A*24:03:02
1/6
A*24:03:03
1/6
A*33:01:01G
1
DQB1*06:02g
1
DQB1*06:04g
1
Build single-locus genotypes
A*24:03:01G+A*33:01:01G
2/3
A*24:03:02+A*33:01:01G
1/6
A*24:03:03+A*33:01:01G
1/6
DQB1*06:02g+DQB1*06:04g
1
Build final MLGs in target resolution
A*24:03:01G+A*33:01:01G^DQB1*06:02g+DQB1*06:04g
2/3
A*24:03:02+A*33:01:01G^DQB1*06:02g+DQB1*06:04g
1/6
A*24:03:03+A*33:01:01G^DQB1*06:02g+DQB1*06:04g
1/6
