# Supplementary material for: Hapl-o-Mat: open-source software for HLA haplotype frequency estimation from ambiguous and heterogeneous data
Source: BMC Bioinformatics. 2017 May 30;18:284. doi: 10.1186/s12859-017-1692-y (PMC5450239; doi:10.1186/s12859-017-1692-y)
Supplement: Supplementary file 2 — Translation between Typing Resolutions. (PDF 303 kb) [file 12859_2017_1692_MOESM2_ESM.pdf]

Additional File 2 – Translation between Typing Resolutions

In the following, we explain how Hapl-o-Mat translates between typing resolutions. Since the translation of an HLA typing assignment into a different typing resolution can result in additional HLA assignments comprised by the initial typing, HLA assignments are weighted by frequencies. Generally, the given HLA designation encompasses n allelic designations in target resolution. To each of them a fraction of $f_i = 1/n$ is assigned and hence summing up to $f = 1$. If the designation arises from an NMDP code, its frequency might be smaller, $f \leq 1$. The frequencies of comprised HLA designations always sum to the initial frequency, $\sum_i f_i' = f$.

The translation process of a given HLA typing assignment starts with identifying its typing resolution. If the present HLA assignment includes more fields than the targeted resolution, the typing is translated by removing the dispensable fields. For instance, translating the 3 field HLA designation A*01:01:16 to a 2 field target resolution, we discard the third field yielding A*01:01. As the initial assignment with frequency f translates into only one assignment, the resulting frequency is $f_1' = f$.

If the present HLA assignment includes less fields than the targeted resolution, the assignment is expanded. To this end, it is replaced with its enclosed HLA assignments. For example, translating to a 4 field typing resolution, the 3 field HLA assignment A*02:02:01 is expanded to its enclosed 4 field assignments A*02:02:01:01 and A*02:02:01:02. As the initial HLA assignment with frequency f splits into two assignments, the resulting frequencies are $f_1' = f_2' = 0.5f$.

An allelic typing is translated into a g, P, or G group by expanding it to its maximal resolution and looking up the resulting assignments in the group definition. If an assignment is not found, the translation proceeds by reducing it to two fields for groups g and P and to three fields for group G. Translating the 2 field HLA assignment A*02:02 to G groups, it is expanded to its enclosed assignments in maximal resolution yielding A*02:02:01:01, A*02:02:01:02, A*02:02:02, and A*02:02:03 with frequencies $f_i' = 0.25f$. Next, HLA assignments are summarized to G groups. The HLA assignments A*02:02:01:01 and A*02:02:01:02 belong to group A*02:02:01G. Since two assignments enter this group, its resulting frequency is $f_1' = (0.25 + 0.25)f = 0.5f$ with f being the initial frequency. As the other two assignments do not belong to a G group, their typing resolution is reduced (or in this case retained) to 3 fields yielding A*02:02:02 and A*02:02:03 with frequencies $f_2' = f_3' = 0.25f$. Thus, the translation of the 2 field typing A*02:02 to G groups, yields A*02:02:01G, A*02:02:02, and A*02:02:03 with frequencies $f_1' = 0.5f$, $f_2' = 0.25f$, and $f_3' = 0.25f$, respectively.

A translation from a group is accomplished by replacing it with its enclosed HLA assignments in the target resolution. Translating the P group A*01:83P with frequency f to two field resolution, it is first expanded to its enclosed assignments A*01:83:01 and A*01:83:02 with respective frequencies $f_i' = 0.5f$. The resulting HLA assignments are translated to two field resolution, both yielding the same assignment A*01:83. The resulting frequency is $f_1' = (0.5 + 0.5)f = f$.

A complete example of data preparation including translation between typing resolutions is given in Additional File 1.
