# Supplementary material for: Hapl-o-Mat: open-source software for HLA haplotype frequency estimation from ambiguous and heterogeneous data
Source: BMC Bioinformatics. 2017 May 30;18:284. doi: 10.1186/s12859-017-1692-y (PMC5450239; doi:10.1186/s12859-017-1692-y)
Supplement: Supplementary file 3 — Methods. (PDF 609 kb) [file 12859_2017_1692_MOESM3_ESM.pdf]

Additional File 3 - Methods

Observables

We compared haplotype frequencies by using different observables. All observables were based on the absolute difference between estimated and reference frequency, h_k and h'_k, of haplotype k, $\delta_k = |h_k - h'_k|$. The first observable was the Manhattan distance $d = \sum_k \delta_k$ which in contrast to e.g. the Euclidean distance does not weight the frequency difference yielding an unbiased measure for deviation between estimated and reference frequencies. For normalized frequencies holds $d \in \{d \in \mathbb{R} | 0 \leq d \leq 2\}$. A further observable was the maximal absolute difference $\Delta = \max(\delta_k)$ between estimated and reference frequencies [1]. We also used the rank ρ of the first reference haplotype with a relative deviation $\delta_k / h'_k > 0.05$ as an observable [1]. For the EM algorithm, the default value of the stop criterion was $\varepsilon = 1/2N$ with N being the size of the population sample. Frequencies can differ on scales smaller than this value. Since HWE is a necessary condition for applying the EM algorithm, we estimated deviation from it via the effect size statistic W_n [2] summed over all loci l

$$W_n = \sum_{l=1}^{s} W_{n,l}$$

where s is the number of loci. In contrast to testing for deviation from HWE using p-values, the effect size statistic gives reliable results even for large sample sizes [3]. For populations with $W_n \ll 0.3$ [4], we assume no significant deviation from HWE.

Artificial Populations

The artificial populations were based on g group resolution haplotypes with an estimated positive frequency from $N = 1{,}825{,}721$ individuals. The individuals with self-assessed German origin from the German DKMS donor center were typed for the genes HLA-A, -B, -C, -DRB1, -DQB1, and -DPB1. The percentage of typing resolutions is listed in Supplementary Table 1. The effect size statistic averaged over all loci was $W_n = 0.0013$ indicating no major deviation from HWE. We used Hapl-o-Mat to estimate haplotype frequencies from this data. As a consequence of expanding NMDP codes, 233 genotypes split into more than 10^5genotypes each and were discarded from analysis.

Locus	NMDP codes [%]	G groups [%]	2 fields [%]	3 fields [%]	4 fields [%]
A	30.5	64.5	3.1	0.4	1.5
B	27.7	57.9	5.2	1.8	7.4
C	31.2	63.2	4.1	1.0	0.5
DQB1	20.0	1.4	7.0	27.6	44.0
DRB1	8.4	7.5	11.7	24.9	47.6
DPB1	31.7	7.8	8.9	29.6	21.9
Average	24.9	33.7	6.7	14.2	20.5

Supplementary Table 1 Percentage of typing resolutions per locus for N=1,825,721 individuals with self-proclaimed German origin. The last line gives the arithmetic mean over all loci.

As detailed below, we considered two models to build artificial genotypes with known haplotype frequency distribution from these results. The process of building artificial populations and reproducing their haplotype frequencies is illustrated in Supplementary Figure 1.

The first artificial population was built by combinatorial construction of diplotypes from all possible combinations of the $1{,}000$ most frequent German haplotypes with replacement. Diplotype frequencies were obtained from haplotype frequencies using the formula

$$g(h_1h_2) = \begin{cases} 2h_1h_2\,, & \text{if } h_1 \neq h_2 \\ {h_1}^2\,, & \text{else} \end{cases}.$$

If several diplotypes yielded the same genotype, the corresponding diplotype frequencies were summed up to the genotype frequency. This resulted in 445,210 different genotypes with associated genotype frequency. An artificial population comprising individuals having these genotypes in proportions according to their frequencies was constructed. The number of individuals of the same genotype was obtained as the rounded integer result of the division of the associated genotype frequency by the minimum genotype frequency. The number of individuals in this population was $N = 15{,}953{,}394$. Population haplotype frequencies were computed by counting haplotypes used in genotype construction and normalizing their number. These frequencies differed from the initial haplotype frequencies, due to the integer-valued genotype numbers. This population model included the complete set of genotypes which can be constructed from a given set of haplotypes. Because of that, we expect a rather accurate reproduction of the initial haplotype frequencies from applying the EM algorithm.

Regarding the second population model, we considered the $10{,}000$ most frequent haplotypes from the real data and thereof drew a sample of $100{,}000$ haplotypes with replacement according to the real frequency distribution. Population haplotype frequencies were computed by counting drawn haplotypes and normalizing their number. Genotypes were constructed by random combination of two haplotypes a time from the sample without replacement. This way, we created an artificial population consisting of $N = 50{,}000$ individuals including $41{,}489$ different genotypes. Genotype numbers were obtained by counting the resulting genotypes.

Using Hapl-o-Mat, we estimated haplotype frequencies from the artificial genotype data. In order to avoid sampling errors, we considered full populations as input. The estimated haplotype frequencies were compared to the haplotype frequencies of the artificial populations using distance d, maximal absolute difference Δ, and rank ρ.

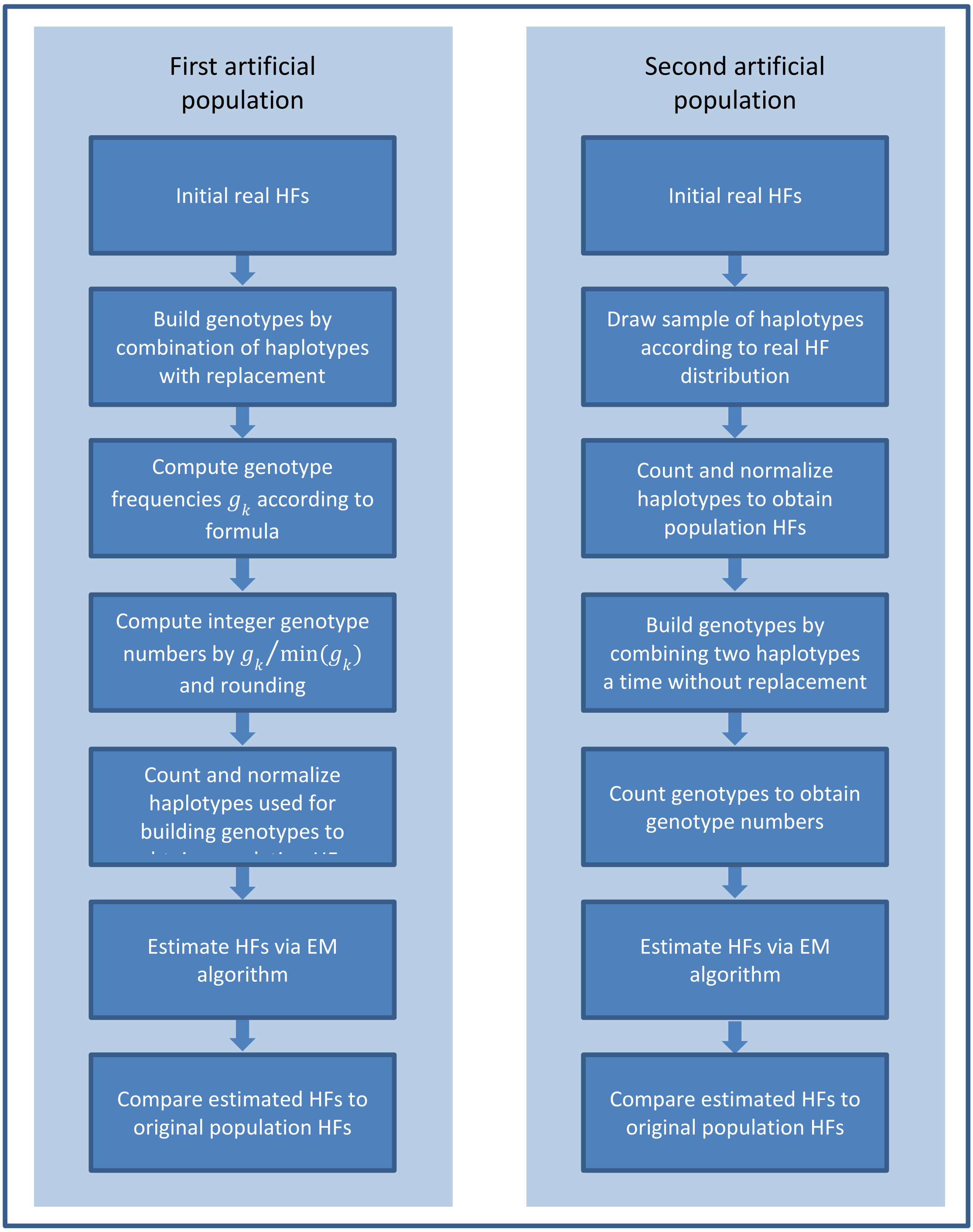

Supplementary Figure 1 Construction of artificial populations for validating Hapl-o-Mat. We used two different approaches to build artificial genotypes with known haplotype frequency distribution. From the artificial genotype data, haplotype frequencies (HFs) were estimated using Hapl-o-Mat. Afterwards, Hapl-o-Mat was validated by comparison between estimated and original population haplotype frequencies

References

1. Schmidt AH, Solloch UV, Pingel J, Baier D, Böhme I, Dubicka K, Schumacher S, Rutt C, Skotnicki AB, Wachowiak J *et al*: **High-resolution human leukocyte antigen allele and**

haplotype frequencies of the Polish population based on 20,653 stem cell donors. *Hum Immunol* 2011, **72**(7):558-565.

2. Klitz W, Stephens JC, Grote M, Carrington M: **Discordant patterns of linkage disequilibrium of the peptide-transporter loci within the HLA class II region**. *Am J Hum Genet* 1995, **57**(6):1436-1444.
3. Eberhard HP, Feldmann U, Bochtler W, Baier D, Rutt C, Schmidt AH, Muller CR: **Estimating unbiased haplotype frequencies from stem cell donor samples typed at heterogeneous resolutions: a practical study based on over 1 million German donors**. *Tissue Antigens* 2010, **76**(5):352-361.
4. Cohen J: **Statistical Power Analysis for the Behavioral Sciences**: Taylor & Francis; 2013.
